# The impact of polygenic risk for alzheimer’s disease on neurotransmitter-related grey matter atrophy in the alzheimer continuum

**DOI:** 10.1007/s10072-025-08393-3

**Published:** 2025-08-25

**Authors:** Riccardo Manca, Micaela Mitolo, Maria Giulia Bacalini, Sabina Capellari, Annalena Venneri

**Affiliations:** 1 Department of Life Sciences, Brunel University London, Uxbridge, UK; 2 Department of Medicine and Surgery, University of Parma, Parma, Italy; 3 IRCCS Istituto delle Scienze Neurologiche di Bologna, Bologna, Italy; 4 Department of Biomedical and Neuromotor Sciences (DIBINEM), University of Bologna, Bologna, Italy

**Keywords:** Alzheimer’s disease, Neurotransmitters, Polygenic risk, MRI, Grey matter, Serotonin, Dopamine, APOE

## Abstract

Alzheimer’s disease (AD) pathology has been recently shown to accumulate in multiple brainstem nuclei in pre-cortical disease stages. However, the impact of neurotransmission alterations on brain atrophy and their genetic correlates in AD remain unexplored. This study investigated (1) associations between grey matter (GM) loss and uptake values of PET/SPECT ligands tracing concentration of multiple neurotransmitter receptors/transporters and pathways; (2) the impact of AD polygenic risk scores (AD-PRSs) on such associations along the AD continuum. T1-weighted MRI scans, genetic and clinical data were selected for 800 ADNI participants: 203 cognitively unimpaired older adults (CU), 442 with mild cognitive impairment (MCI) and 155 with AD. JuSpace was used to calculate correlations between GM volume (GMV) and the concentration of several neurotransmitters. Two PRSs, with (AD-PRS) and without APOE (AD-PRS_noAPOE_), were investigated as predictors of the strength of correlation between GMV and neurotransmitters in general linear models. In both patient groups, atrophy was negatively associated with serotoninergic and dopaminergic receptors/transporters. In the whole sample, both PRSs were associated with the strength of correlation between GMV and different serotonin receptors and fluorodopa uptake. The pattern of associations was replicated in participants with evidence of amyloid pathology. GM loss in AD may be particularly affected by the alterations in serotoninergic and in presynaptic dopaminergic activity that are known to influence functioning of medio-temporal and frontal cortices. Such alterations appear to be driven by higher AD-PRS values. Investigating further neurotransmitter-related neural alterations may help clarifying neuropathological changes in pre-clinical AD and response to treatments.

**Supplementary Information:**

The online version contains supplementary material available at 10.1007/s10072-025-08393-3.

## Introduction

Sporadic Alzheimer’s disease (AD) presents with complex and heterogenous neural alterations across patients. Neuroimaging studies have shown that AD-related grey matter (GM) atrophy can manifest in different patterns, not necessarily characterised by medio-temporal volume loss [1, 2]. Groups of AD patients classified based on GM atrophy patterns present with different demographic and genetic risk factors [1]. For instance, limbic predominant GM atrophy was associated with a higher prevalence of the ε4 allele of the apolipoprotein E (APOE). APOE ε4 is the single nucleotide polymorphism (SNP) most strongly associated with sporadic AD risk and the most studied with respect to its effects on GM degeneration in AD [3].

A growing body of evidence has accumulated on the involvement of non-APOE SNPs that could be contributing to increase risk of neurodegeneration and of AD. SNPs across the whole genome have been investigated in combination by generating either polygenic hazard scores (PHS) [4] or polygenic risk scores (PRS) [3, 5] that are increasingly used as quantitative indices of risk for any given disease (or for specific traits). These scores are calculated as weighted sums of SNPs by using the results of genome-wide association studies (GWASs) to apply the weights to individual genome data of participants included in a target sample different from that used in the discovery GWAS [6]. Specific PRSs can also be generated by selecting SNP sub-sets [5, 7].

AD-PRSs have been used to predict a range of clinical outcomes [8, 9] including neural alterations [10]. GM volume (GMV) has been the parameter more extensively investigated, especially in selected medio-temporal areas. Across the AD continuum, higher AD-PRS and AD-PHSs have been found associated with smaller volumes (cross-sectionally) and greater GM loss (longitudinally) in the hippocampus and in the entorhinal cortex [8–14], even when the weight of the APOE alleles was excluded [9]. Voxel-based investigations found that the PHS by Desikan et al. [4] was associated with smaller bilateral hippocampal and amygdala GM volumes only in people diagnosed with mild cognitive impairment (MCI), but not in cognitively unimpaired (CU) older adults [15]. This association was not significant after stratifying the sample by APOE genotype, a finding consistent with the strong effect of APOE ε4 on medio-temporal GMV observed also by Lupton et al. [11]. A study using a more up-to-date AD-PRS [16] found a negative impact on voxel-based GM atrophy that extended beyond the medio-temporal lobe to posterior cingulate cortex, an area that undergoes volumetric loss in the early stages of sporadic AD [17].

This genetic modulation of GM alterations has been almost exclusively investigated in cortical areas, despite recent evidence of early pre-cortical AD pathological changes, i.e., accumulation of tau tangles, in brainstem nuclei [18]. These small brain structures, which are involved in the production of various neurotransmitters, and the potential impact of AD pathology on their integrity remain difficult to investigate in vivo due to the current limitations in neuroimaging technology. However, reduced MRI-derived integrity of the ventral tegmental area was observed in CU older adults who progressed to MCI compared with those who showed no signs of cognitive decline [19]. Consistently, Sala et al. [20] have shown, using molecular neuroimaging, that AD is associated with metabolic alterations in the mesocorticolimbic, but not in the nigro-striatal dopaminergic pathway. Moreover, increased plasma levels of phosphorylated tau have been found associated with reduced integrity of the locus coeruleus of CU older adults [21].

The role of different neurotransmitter systems in AD has regained interest recently with the availability of refined methodology. Given that neurons of the brainstem nuclei project extensively across the brain, the investigation of brain connectivity seems to be crucial to explain the evolution of AD pathology. Indeed, in patients with AD, the spread of tau aggregates is observed across functionally connected brain areas [22] and Aβ accumulation is a partial mediator of this process [23]. Such process may involve tau fragments spreading from neuron to neuron via synapses due to upregulation of synaptic transmission induced by Aβ [24]. Therefore, it is possible that AD-related tau pathology may start in the brainstem and spread subsequently to cortical areas. Genetic risk for AD is likely to play a role in such a process.

Different AD-PRSs have been associated with alterations in a variety of AD fluid biomarkers, including phosphorylated tau, both in blood [25] and in the cerebrospinal fluid (CSF; [26, 27]). Due to the nature of PRS measures, however, it is not possible to establish whether one or multiple interacting pathways may be influencing the earliest AD neural changes. Based on findings of previous GWASs, it is plausible to hypothesise that multiple biological mechanisms, including immune system and lipid metabolism dysfunction, may be contributing factors [4, 28]. Moreover, functional analysis performed by Jansen et al. [28] showed the strongest genetic risk factors for AD are expressed in various brain cell types, including predominantly microglia, but also dopaminergic and serotoninergic neurons.

These novel findings suggest that dysfunction in multiple neuromodulatory systems stemming from the brainstem and innervating many cortical and subcortical areas represents one of the earliest neural alterations in AD. Considering the impact of genetic risk factors in AD, it is possible that AD-PRSs may be associated with GM atrophy preferentially in areas influenced by specific neurotransmitter pathways. However, this potential relationship remains to be clarified. For this reason, the aims of this study were to investigate:

1. Whether GM atrophy in people along the AD continuum (either with MCI or AD) was associated with the cerebral concentration of neurotransmitters, i.e. neurotransmitter-related GM atrophy;
2. The impact of AD-PRS (either including or excluding the weight of APOE) on the association between GMV and cerebral concentration of neurotransmitters.

## Methods

### Participants

Data from the Alzheimer’s Disease Neuroimaging Initiative (ADNI) database (adni.loni.usc.edu) were used for this investigation. The ADNI was launched in 2003 as a public-private partnership, led by Principal Investigator Michael W. Weiner, MD. The primary goal of ADNI has been to test whether serial magnetic resonance imaging (MRI), positron emission tomography (PET), other biological markers, and clinical and neuropsychological assessment can be combined to measure the progression of MCI and early AD. For up-to-date information, see www.adni-info.org. The ADNI protocol received ethics approval at each participating institution and participants provided written informed consent.

A sample of 812 participants with genotyping data carried out by ADNI using the Illumina OmniExpress array [29] were screened and included in this study based on availability of demographic, diagnostic, cognitive and structural MRI data. Participants were excluded if any of the abovementioned assessments was unavailable or if they had a history of psychiatric diseases.

Participants with no evidence of cognitive deficits at all the available time points were identified as the CU group. Six of these participants were excluded due to the presence of psychiatric symptoms. Five participants were excluded as they had a sibling in the sample to avoid biases due to relatedness [30]. One participant was removed due to missing MRI data. As a result, 800 participants were retained for analyses: 203 CU, 442 MCI and 155 AD.

A set of neuropsychological tests was also extracted to characterise the cognitive profile of the two patient groups: Mini Mental State Examination, Clock Drawing Test (drawing and copy), Logical Memory Test (immediate and delayed recall), Category Fluency Test (animals) and Trail Making Test (part A).

### PRS calculation

Two PRSs for AD risk were calculated: one with (AD-PRS) and one without APOE (AD-PRS_noAPOE_). The same procedure used by Manca et al. [16] was followed. Briefly, PLINKv2.0 was used to pre-process the genetic data provided by ADNI by setting quality control parameters at imputation information content > 0.9, minor allele frequency > 0.05 and Hardy-Weinberg equilibrium mid-p-value > 10^−6^. A total of 1.3 million single nucleotide polymorphisms (SNPs) were retained after quality control. The “GENESIS” R package [31] was used to generate 10 genetic principal components (PCs) with PC-AiR [32] and to estimate relatedness with PC-Relate [33]. The 10 PCs were used as covariates in all statistical analyses. To calculate the AD-PRS_noAPOE_, the whole APOE region was excluded using the hg19 coordinates chr19 from 44 400 000 to 46 500 000 [10].

Summary statistics from the GWAS by Jansen et al. [28] were used to generate the PRSs. SNPs with imputation information content scores < 0.9 and duplicates were removed. To our knowledge, participants in ADNI were not included in the discovery GWAS used to calculate PRSs. PRSs were generated using a Bayesian approach with continuous shrinkage priors [34]. After accounting for linkage disequilibrium using the 1000 Genomes EUR samples, 455,028 SNPs were retained for AD-PRS and 454,638 SNPs for AD-PRS_noAPOE_. Bayesian posterior effect sizes were calculated with the shrinkage parameter inferred using the PRS-CS-auto method. Lastly, PRSs were calculated in PRSice v2 [6] without pruning at 10 *p*-value thresholds: 5 × 10^−8^, 1 × 10^−6^, 1 × 10^−5^, 0.0001, 0.001, 0.01, 0.05, 0.1, 0.5 and 1. PRSs were z-transformed to be used in all statistical analyses.

### Structural MRI data pre-processing

T1-weighted MRI data were acquired as specified in the ADNI MRI protocol [35]. Both scans acquired at 1.5T (*n* = 248) and 3 T (*n* = 552) were included in order to maximise the sample size. Images were reoriented to the bi-commissural axis and subsequently pre-processed using the CAT12 (version 1830) toolbox (https://neuro-jena.github.io/cat/) for SPM12 (https://www.fil.ion.ucl.ac.uk/spm/software/spm12/) running on MATLAB R2021a (the MathWorks, Inc., Natick, MA, USA). First, reoriented images were segmented into GM, white matter (WM) and CSF tissue maps. Second, GM maps were normalised to a standard ICBM template in the MNI space and modulated. Third, normalised GM maps were smoothed with an 8 mm full-width at half maximum Gaussian kernel. Global volumes of GM, WM and CSF were quantified using SPM12 and the total intracranial volume (TIV) of each participant was calculated by summing the volume values of the 3 tissue classes.

Subsequently, the JuSpace toolbox (version 1.5) was used to investigate associations between GMV and specific neurotransmitter systems [36]. Smoothed GM maps were parcelled in 119 regions using the Neuromorphometrics Atlas (MICCAI 2012 Grand Challenge and Workshop on Multi-Atlas Labeling). The same parcellation was applied to the following 14 PET/SPECT atlases included in JuSpace: the 5-hydroxytryptamine 1a (5-HT_1a_), 1b (5-HT_1b_), 2a (5-HT_2a_) and 4 (5-HT_4_) serotonin receptors, the serotonin transporter (SERT), the D1 and D2 dopamine receptors, the dopamine transporter (DAT), the overall brain dopaminergic system quantified by Fluorodopa (FDOPA) uptake, the Gamma-Aminobutyric Acid a (GABAa) receptor, the noradrenaline transporter (NAT), the vesicular acetylcholine transporter (VAChT), the N-methyl-D-aspartate (NMDA) and the metabotropic glutamate type 5 (mGLUR5) receptors for glutamate. These atlases were used to investigate the widest range of neurotransmitter systems compatibly with current availability in JuSpace.

### Statistical analyses

Demographic, clinical and cognitive profiles were compared across the 3 groups using either ANOVA or Kruskal-Wallis tests, for continuous variables normally and not normally distributed, respectively, or χ^2^, for categorical variables. Bonferroni’s correction for multiple comparisons was applied to the analyses of the above variables by using a significance threshold of *p* = 0.0038 (i.e., 0.05/13).

JuSpace was used to calculate individual Fisher’s Z-transformed Spearman correlation coefficients between regional GMV and the various neurotransmitter maps included in the toolbox and compared across groups using Option 5 in 3 separate models to investigate aim #1: CU vs. MCI, CU vs. AD and MCI vs. AD. Significance *p*-values (FDR-corrected) were determined using 10,000 permutations. Significant negative differences are assumed to indicate that GMV loss in patients, compared with CU (and in the AD compared with the MCI group), is stronger in brain regions with higher distribution of the investigated neurotransmitters, while positive differences indicate GMV loss in regions with lower neurotransmitter distribution.

In order to assess GM loss along the AD continuum, VBM analysis was carried out to compare GM maps across groups using an ANOVA model and 3 independent-samples *t*-tests as *post hoc* analyses: CU vs. MCI, CU vs. AD and MCI vs. AD. All models were implemented in SPM12 and included age, sex, TIV and MR field strength as covariates and with a cluster-forming threshold of *p* < 0.05 with family-wise error (FWE) correction for multiple comparisons.

JuSpace was also used to calculate individual Fisher’s Z-transformed Spearman correlation coefficients between GMV and each neurotransmitter for all participants using Option 8. This option is used to determine whether individual coefficients deviate from the null distribution (exact p-values determined using 10,000 permutations). General linear models were subsequently used to assess whether the relationship between GMV and neurotransmitter distribution was influenced by polygenic risk for AD (aim #2). Individual Fisher’s Z-transformed Spearman correlation coefficients between GMV and each neurotransmitter were treated as dependent variables and PRSs as independent variables. Ten models were run for each PRS calculated at different thresholds including 10 genetic PC, age, sex, TIV and MR field strength as covariates. Analyses were carried out on the whole sample and in the 3 diagnostic groups separately. In line with a previous publication [37] that adopted a similar approach, a negative association between a PRS and GMV-neurotransmitter correlation coefficients is interpreted by assuming that people with higher PRS values would show lower GMV in areas where the density of a specific neurotransmitter is higher. Conversely, a positive association would indicate that people with higher PRSs should present with lower GMV in areas where the neurotransmitter density is lower.

Complementary VBM regression models were used to investigate the spatial pattern of association between both PRSs (10 models for each PRS calculated using different thresholds specified above) and GMV (*p* < 0.05 with FWE correction for multiple comparisons). These models included 10 genetic PCs, age, TIV, sex and MR field strength as covariates.

All participants, irrespectively of biological evidence of AD pathological changes, were initially retained in order to maximise the sample size for PRS analyses. Subsequently, two additional sensitivity analyses were carried out to assess whether any observed associations were primarily detectable in participants with evidence of amyloid beta positivity (Aβ+) (either on CSF or PET examinations) across the AD continuum:

1) JuSpace and VBM models were replicated to compare 79 Aβ+ CU, 274 Aβ+ MCI and 128 Aβ+ AD participants with 105 Aβ- CU participants and to compare Aβ+ groups;

2) General linear models (for GMV-neurotransmitter correlation coefficients) and VBM regression models were replicated in a subsample of 481 Aβ+ participants, irrespectively of diagnostic status (i.e., 79 CU, 274 MCI and 128 AD). Considering the large number of analyses, the standard significance threshold (*p* = 0.05) was Bonferroni-corrected based on the number of PET atlases (*n* = 14), i.e. *p* = 0.003571.

Considering the central role given to Aβ status in AD diagnosis, additional analyses (Kruskal-Wallis and two-sample VBM t-test) were carried out to compare both clinical and GM volumetric profiles, respectively, between Aβ- and Aβ+ patient groups (MCI and AD separately).

All statistical analyses were run in IBM SPSS Statistics version 26 (IBM, Chicago, IL, USA).

## Results

### Clinical profiles

Participants were matched for years of education and TIV, while the AD group was significantly older than the other groups (Table 1). The CU group had a significantly higher proportion of female participants than the MCI group, but this difference did not survive Bonferroni’s correction for multiple comparisons. Both patient groups had lower cognitive scores, compared with CU participants, across all tests except for the copy trial of the Clock Drawing test where the MCI group had scores similar to the CU group. Participants with AD performed worse than people with MCI on all tests.

**Table 1:** Demographic and clinical profile of the participant samples. All summary statistics are mean (standard deviation) unless otherwise specified (Bonferroni-corrected *p* = 0.0038)

Variables	CU (*n* = 203)	MCI (*n* = 442)	AD (*n* = 155)	F	*p*
*Demographic*					
Age	73.8 (5.8)^a^	73.3 (7.9)^a^	76.9 (8.0)	25.9^b^	< 0.001
Education	16.5 (2.6)	16.0 (2.8)	16.0 (2.9)	4.9^b^	0.085
Sex (%, F/M)	52.2/47.8	41.4/58.6	44.5/55.5	6.6^c^	0.037
*Cognitive*					
MMSE (total score)	29.1 (1.2)^a, d^	27.9 (1.8)^a^	21.4 (5.0)	343.7^b^	< 0.001
CDT (Drawing)	4.7 (0.5)^a, d^	4.5 (0.7)^a^	3.3 (0.9)	140.0^b^	< 0.001
CDT (Copy)	4.9 (0.3)^a^	4.8 (0.5)^a^	4.1 (1.3)	90.7^b^	< 0.001
LMT (Immediate recall)	14.7 (2.9)^a, d^	10.0 (1.0)^a^	4.2 (3.4)	395.1^b^	< 0.001
LMT (Delayed recall)	13.7 (3.2)^a, d^	7.7 (3.8)^a^	1.9 (3.1)	458.0^b^	< 0.001
CFT (Animals)	21.2 (5.6)^a, d^	18.2 (5.0)^a^	11.0 (5.2)	232.8^b^	< 0.001
TMT-A (seconds)	33.2 (10.4)^a, d^	38.3 (15.4)^a^	68.0 (43.6)	116.5^b^	< 0.001
*Imaging and biomarkers*					
TIV	1433.2 (137.3)	1459.4 (138.1)	1454.2 (165.7)	2.5	0.080
Aβ status (%, +/-/missing)	38.9/51.7/9.4^a, d^	62.0/33.5/4.5^a^	82.6/8.4/9.0	84.7^c^	< 0.001
APOE (%)				95.0^c^	< 0.001
*ε4ε4*	1.5^a, c^	5.7^a^	18.1		
*ε3ε4*	22.2^a, c^	31.7^a^	49.0		
*ε3ε3*	62.6^a, c^	52.3^a^	27.7		
*ε2ε4*	0.5	2.3	2.6		
*ε2ε3*	13.3^a, c^	7.9^a^	2.6		
*ε2ε2*	0.0	0.2	0.0		

Aβ: Amyloid beta, AD: Alzheimer’s disease, APOE: Apolipoprotein E, CDT: Clock Drawing test, CFT: Category Fluency test, CU: Cognitively unimpaired, F: Female, LMT: Logical Memory test, M: Males, MCI: Mild cognitive impairment, MMSE: Mini Mental State Examination, TMT-A: Trail Making Test– part A

^a^ Significant difference compared with the AD group

^b^ Kruskal-Wallis test

^c^ χ^2^ test

^d^ Significant difference compared with the MCI group

The proportion of people with evidence of positive Aβ biomarkers was increasingly higher along the AD clinical continuum: 38.9% in CU, 62.0% in MCI and 82.6% in AD. Moreover, people with AD were significantly more likely to be APOE ε4 carriers (either homozygous or heterozygous) and less likely to have either ε3ε3 or ε3ε2 genotypes than the other groups. The same pattern of genotype distribution was found in the MCI compared with the CU group.

### GM atrophy and associations with neurotransmitter distribution

**Fig. 1:**
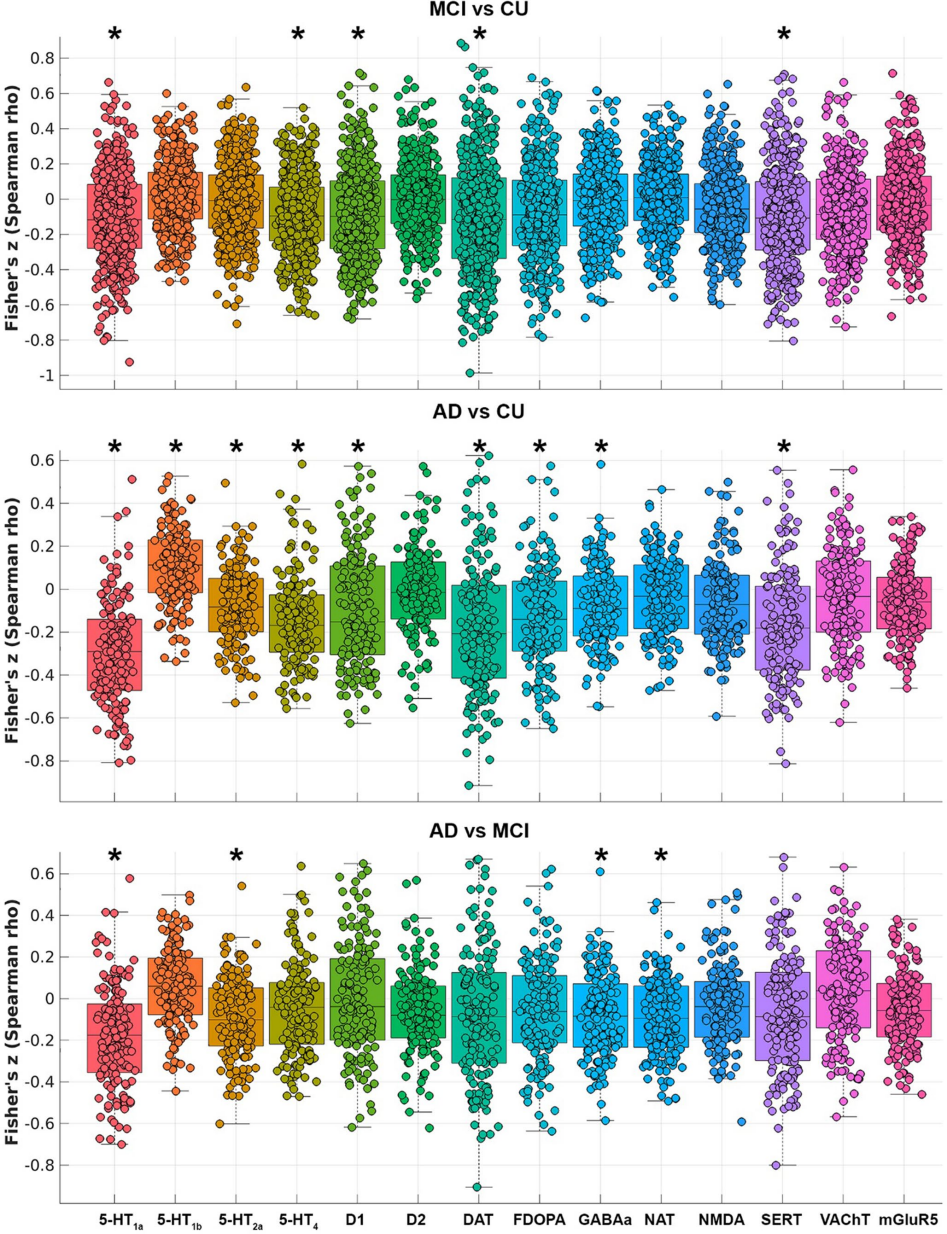
Associations between grey matter atrophy in MCI and AD groups and *a priori* density values of neurotransmitters (significant results are indicated by an asterisk)

GM atrophy in the MCI group was mainly associated with the distribution of dopaminergic and serotoninergic receptors (5-HT_1a_, 5-HT_4_, D1) and transporters (DAT and SERT) (Fig. 1). Similar findings were also observed for the AD group who also expressed additional associations between GM atrophy and the distribution of all the serotonin receptors, FDOPA uptake and the GABAa receptor. While most associations were negative, i.e. the higher the *a priori* neurotransmitter density values the greater the GM atrophy, a positive association was found for the 5-HT_1b_ receptor, i.e. the higher the *a priori* 5-HT_1b_ receptor density values the lower the GM atrophy. The comparison between patient groups, instead, highlighted that greater GM atrophy in people with AD was associated with higher density of serotoninergic and GABAa receptors and of the noradrenergic transporter (i.e. NAT).

### Patterns of GM atrophy

**Fig. 2:**
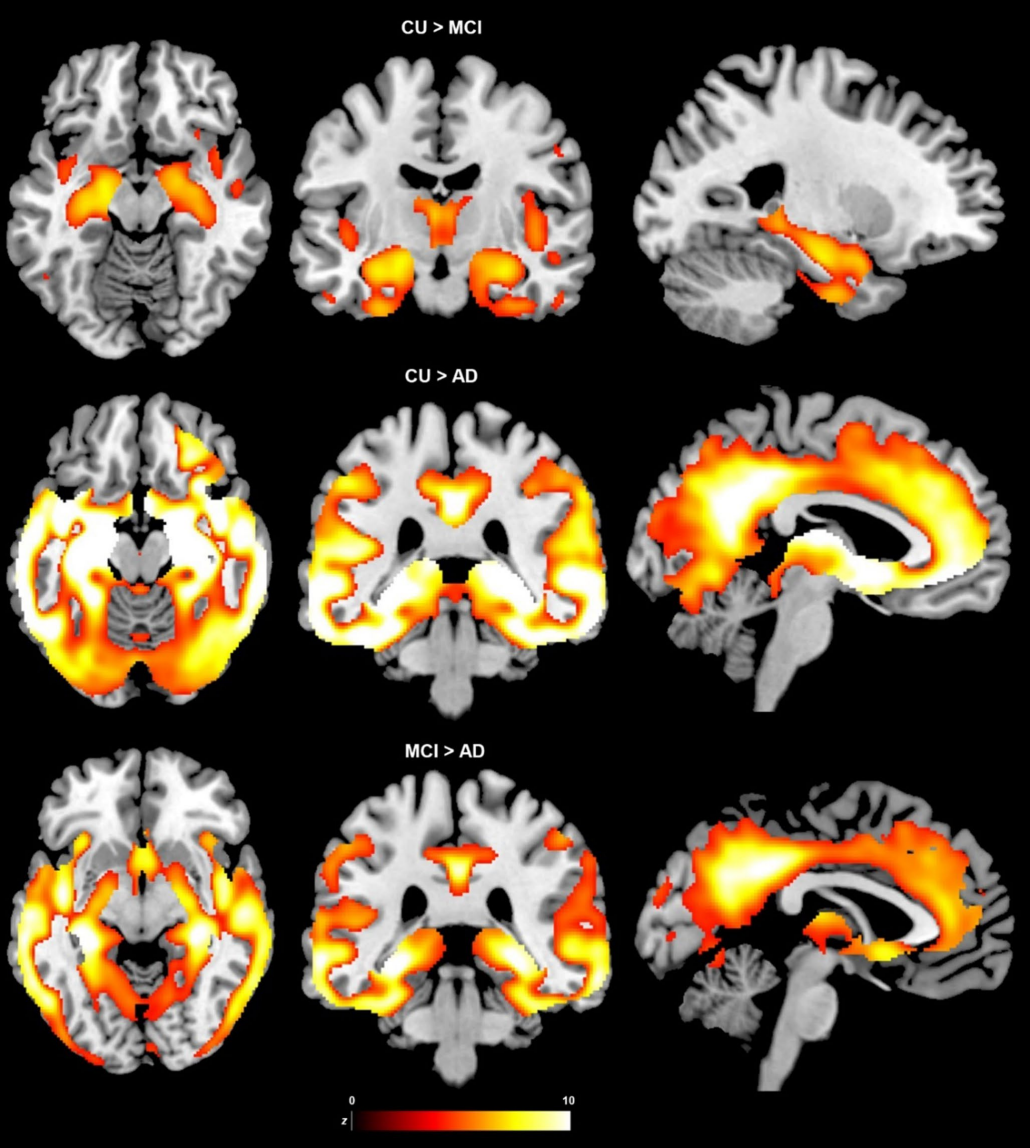
Grey matter atrophy along the AD continuum (*p* < 0.05 FWE-corrected for multiple comparisons)

VMB ANOVA analysis revealed a pattern of widespread GMV differences across groups (Supplementary Fig. 1). In detail, GM atrophy was evident in both patient groups compared with CU participants: primarily in medio-temporal, inferior and lateral temporal and thalamic areas in the MCI group, and more widespread throughout cortical and subcortical areas in the AD group (Fig. 2). People with AD had a more severe GM atrophy pattern than those with MCI, even in medio-temporal areas.

### Association between PRSs and neurotransmitter-GMV correlation coefficients

**Fig. 3:**
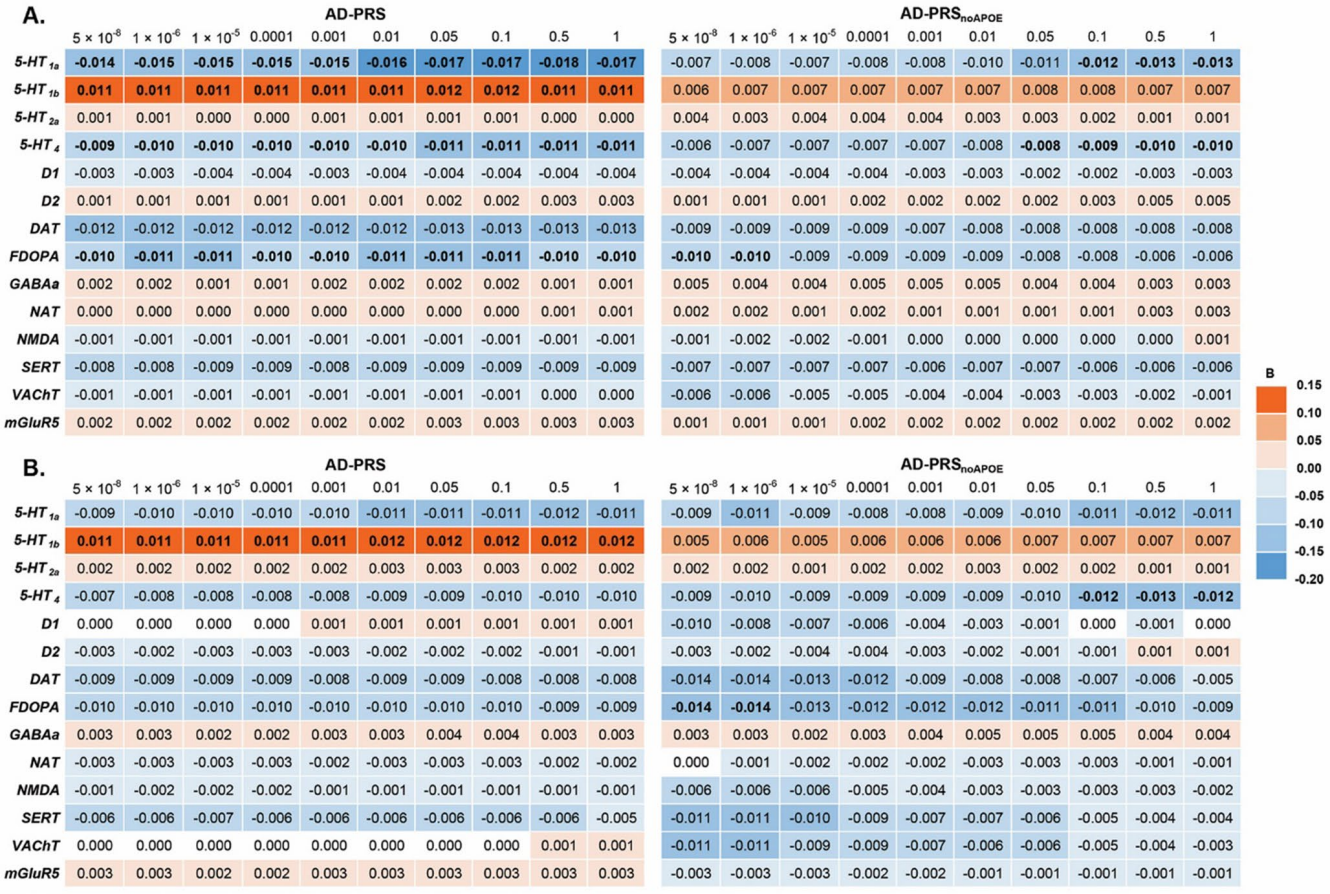
Associations between both PRSs and GMV-neurotransmitters correlation coefficients **A** in the whole sample (*n* = 800) and **B** in the subsample of Aβ + participants (*n* = 481). Significant associations are highlighted in bold

In the whole sample, the AD-PRS was negatively associated with coefficients of correlation between GMV and 5-HT_1a_, 5-HT_4_ and FDOPA uptake and positively associated with GMV-5-HT_1b_ correlation coefficients (Fig. 3). The AD-PRS_noAPOE_ was also negatively associated with coefficients of correlation between GMV and 5-HT_1a_, 5-HT_4_ and FDOPA uptake.

For AD-PRS, the above-mentioned associations were detected irrespectively of the significance threshold used to generate the PRS. By contrast, when the AD-PRS_noAPOE_ was generated with conservative thresholds (i.e. by including only the SNPs most strongly linked to AD risk), associations were observed with FDOPA uptake-GMV correlation coefficients. The AD-PRS_noAPOE_ calculated with less conservative threshold (i.e. including most if not all DNA, but excluding the APOE region) was associated with 5-HT-GMV correlation coefficients (Fig. 3).

### Spatial patterns of PRS-GMV associations

VBM regression models in the whole sample showed that higher AD-PRS values were associated with lower GMV in four clusters located in bilateral medio-temporal areas, primarily centred in the hippocampi, the right fusiform gyrus and (only for the 5 PRSs calculated using the most conservative thresholds) in the right precuneus and posterior cingulate cortex (Supplementary Fig. 2). Similar findings were observed for the AD-PRS_noAPOE_, with negative associations in bilateral medio-temporal (more predominantly in the left hippocampus) and in left inferior temporal areas (Supplementary Fig. 3).

### Sensitivity analyses

In Aβ+ MCI and Aβ+ AD groups, results on the associations between GM atrophy (compared with the Aβ- CU group) and the distribution of neurotransmitters, especially with serotoninergic receptors and transporter, were largely replicated (Supplementary Fig. 4). No associations were found between neurotransmitter distribution and GMV between Aβ+ and Aβ- CU groups, possibly due to the lack GMV differences between Aβ+ and Aβ- CU groups.

When Aβ+ patients where compared either with Aβ+ CU participants or with one another, similar results were observed, i.e., GM atrophy primarily associated with the distribution of different serotoninergic receptors and SERT and, to a minor extent, with D2 and DAT (Supplementary Fig. 5).

VBM models confirmed the findings of GM atrophy in bilateral temporal areas in the Aβ+ MCI group and widespread GM atrophy in the Aβ+ AD group compared with Aβ- CU older adults (Supplementary Fig. 6). The same pattern emerged also when Aβ+ patient groups were compared with Aβ+ CU participants (Supplementary Fig. 7).

A few associations between PRSs and neurotransmitter-related GMV survived in the Aβ+ sub-sample: with the 5-HT_4_ receptor, for AD-PRS, and with the 5-HT_4_ receptor and FDOPA uptake only, for AD-PRS_noAPOE_ (Fig. 3).

VBM regression models revealed a consistent pattern of negative associations between the AD-PRS inclusive of APOE and the volume of bilateral medio-temporal right fusiform, precuneus and posterior cingulate areas (Supplementary Fig. 8). Instead, no associations were detected for the AD-PRS_noAPOE_ when analyses were restricted to Aβ+ participants.

Details on the clinical profile of these groups can be found in Supplementary Table 1.

In the MCI sample, Aβ- participants appeared to show milder cognitively deficits than Aβ+ participants, since they presented with higher MMSE and Logical Memory - delayed recall scores. Moreover, they were also slightly younger and less likely to be APOE ε4 allele carriers than Aβ+ patients (Supplementary Table 2). The latter finding emerged also when AD patients with different amyloid statuses were compared (Supplementary Table 3). No significant differences in GMV were found between Aβ+ and Aβ- patients in either clinical groups.

## Discussion

In this study, GM atrophy due to AD was associated primarily with the concentration of dopaminergic and serotoninergic receptors and transporters. This association was evident already at the MCI stage, when GM atrophy was localised in medio-temporal and limbic areas, even though it emerged only for a subset of receptors (i.e. D1, 5-HT_1a_ and 5-HT_4_) and transporters (i.e. DAT and SERT). Higher AD-PRS values were negatively associated with the strength of correlation between GMV and density of FDOPA uptake, 5HT_1a_ and 5HT_4_ receptors and positively with GMV-5HT_1b_ correlation coefficients. Although including the APOE region in the calculation of the AD-PRS led to stronger and more reliable findings across PRS thresholds, similar associations were detected for both AD-PRSs. The same associations were also partially replicated in the sensitivity analysis limited to people with evidence of Aβ+ status.

Overall, these findings suggest that GM atrophy in people along the clinical AD continuum is influenced by AD-PRS and more pronounced primarily in brain areas where the serotoninergic receptors are significantly expressed. Indeed, in a previous investigation of the same sample [16] we observed that higher AD-PRS values were associated with lower GMV primarily in medio-temporal areas, but also in right fusiform, precuneal and posterior cingulate regions, both in the whole sample and in Aβ+ participants. Interestingly, density values for the 5-HT_1a_ and the 5-HT_1b_ receptors are, respectively, extremely high and low in medial temporal areas (i.e. the hippocampus and the entorhinal cortex) [38]. Such divergence in the distributions of the two serotoninergic receptors could explain the finding that the AD-PRS was positively associated with GMV-5HT_1a_ but negatively with GMV-5HT_1b_ correlation coefficients. Density of the 5-HT_4_ receptor, instead, is strongest in lateral temporal cortices and in the inferior parietal lobe. All of these areas are more atrophic in patients with AD who are carriers of the APOE ε4 allele [1], in line with our finding of a stronger and more reliable influence of the AD-PRS inclusive of APOE on GMV-neurotransmitter associations.

In this study, therefore, serotonin emerges as a potential mediator of GM degeneration due to AD. Alterations in serotoninergic neurotransmission and receptor density decreases in temporal areas have been observed in AD in association with behavioural deficits [39]. It is possible that a depletion of serotoninergic inputs to the medio-temporal lobe due to AD-induced neuronal death in the raphe nuclei [18] may hinder neurogenesis in the dentate gyrus, a process that seems to be influenced by the activation of multiple serotoninergic receptors in mice [40]. Verdurand & Zimmer [41] have highlighted that 5HT_1a_ receptor expression can be modified by Aβ plaque accumulation in early disease stages leading to receptor loss in the hippocampus and, consequently, to hippocampal dysfunction. Moreover, pharmacological stimulation of different serotonin receptors (e.g. 5HT_1a_, 5HT_4_ and 5HT_6_) seems to have beneficial effects on cognitive performance [42].

Higher AD-PRSs were also associated with smaller GMV in areas where FDOPA uptake was higher, i.e. especially subcortical nuclei (i.e. the putamen and the caudate) and the dorsolateral prefrontal cortices. Previous investigations have found that FDOPA uptake in the basal ganglia and in the midbrain is preserved in normal ageing [43], while a decline can be detected in frontal areas in cognitively unimpaired older adults [44]. Dopaminergic innervation of the frontal lobes is primarily fostered by the mesocorticolimbic pathway that originates from the VTA. Therefore, it is conceivable that the significant influence of AD-PRS on the FDOPA uptake-GMV association may be driven by neural alterations in mesocorticolimbic dopaminergic neurotransmission. Indeed, previous studies using different neuroimaging techniques have revealed an excess of AD-related damage in the mesocorticolimbic, but not in the nigrostriatal pathway, from very early disease stages in AD [19, 20].

Although the APOE gene plays a major role in determining AD-related neural alterations, also non-APOE SNPs contributed to explain smaller GMV in areas with higher serotonin receptor density values and higher FDOPA uptake. Moreover, this pattern was also evident in Aβ+ participants (60% of the whole sample) along the clinical AD continuum. Fewer associations were detected in the sensitivity analysis, probably because of reduced statistical power. VBM regression models showed that AD-PRS_noAPOE_ was primarily associated with reduced medio-temporal and left inferior temporal GMV. This pattern is more consistent with the distribution of both 5-HT_1a_ and 5HT_4_ receptors, thus suggesting a primary involvement of GM atrophy along serotoninergic pathways. Such associations, however, were only found when the whole sample was investigated and did not survive in the Aβ+ sub-sample analysis. This may explain why fewer associations between the AD-PRS_noAPOE_ and neurotransmitter-related GMV were detected than for the AD-PRS inclusive of APOE.

These findings suggest that, in people with AD, GM atrophy in areas primarily influenced by serotoninergic neurotransmission in medio-temporal and infero-temporal areas, may also be determined by APOE-independent processes. Biological mechanisms suggested by the GWAS [28] used in this study, and confirmed by other studies [45, 46], are various and include immune response, inflammation, endocytosis, and cell migration. Moreover, the results of this study are coherent with the outcome of the functional analysis carried out by Jansen and colleagues [28] who have shown how genetic variants associated with increased AD risk were expressed in dopaminergic and serotoninergic neurons, thus suggesting a potential involvement of both neurotransmitter systems in AD-related pathological changes.

This study has a few limitations. First, the design was cross-sectional and, therefore, any interpretation of the potential causal influence of AD-PRS on GM atrophy mediated by specific neurotransmitter receptors/transporters remains speculative. Indeed, between-group comparisons showed that greater GM atrophy was also correlated with higher density of both serotonin and dopamine transporter distribution in the MCI and AD groups. However, both AD-PRSs were not significantly associated with the strength of correlation between GMV and distribution density of either transporters. Second, the modest sample size may have limited the statistical power of the analyses stratified by diagnosis given the inclusion of a large number of covariates, thus preventing the detection of associations within each diagnostic group. Although previous investigations have found significant associations between AD-PRSs and GMV in cognitively healthy samples with a sample size similar to that of this study [12, 47], this is the first multimodal neuroimaging investigation of this kind of a sample including the whole AD continuum. Therefore, only future studies can test the replicability and generalisability of these findings and clarify the relevance of alterations in specific neurotransmitter systems for GM atrophy due to AD. Third, due to current PET/SPECT tracer availability that was not imputable to this study, the potential role of some neurotransmitters suggested to be dysfunctional in AD (e.g. noradrenaline) could not be assessed. Therefore, it is not possible to clarify whether AD polygenic risk can impact brain morphology mediated by dysfunctions in under-investigated neurotransmitter systems. Fourth, due to limited data availability, the two clinical groups also included a minority of Aβ- participants, i.e., people with no evidence of AD pathological changes. Although this may raise concerns with the accuracy of diagnosis, Aβ+ and Aβ- patients had very similar clinical and neuroimaging profiles. Milder memory deficits were found in Aβ- MCI compared with Aβ+ MCI participants, while Aβ- participants were in general less likely to be APOE ε4 carriers. However, sensitivity analyses confirmed the majority of the results, thus ruling out substantial biases in the findings of this study due to inclusion of Aβ- participants. Further investigations in larger cohorts stratified by Aβ status may help clarifying any potential interaction effects between AD-PRSs and amyloid-related effects on neural alterations in different neurotransmitter pathways.

The novel findings of this study suggest that AD polygenic risk can influence GMV variability associated with both serotonin receptor density and dopamine availability levels and that this effect can be detected along the clinical AD continuum. Neurotransmitter-related GM atrophy could represent a marker to investigate specific hypotheses on AD aetiology, to characterise neural alterations in patients with different clinical phenotypes and to test mechanisms of action of neuroprotective drugs targeting specific neurotransmitters, or more generally to test potential changes in regional disease trajectories following disease modifying treatment.

## Supplementary Information

Below is the link to the electronic supplementary material.

ESM 1 (DOCX 3.57 MB)

## Data Availability

All ADNI data are available at adni.loni.usc.edu.
