# Supplementary material for: The impact of polygenic risk for alzheimer’s disease on neurotransmitter-related grey matter atrophy in the alzheimer continuum
Source: Neurol Sci. 2025 Aug 25;46(11):5791–803. doi: 10.1007/s10072-025-08393-3 (PMC12537760; doi:10.1007/s10072-025-08393-3)
Supplement: Supplementary file 1 — (DOCX 3.57 MB) [file 10072_2025_8393_MOESM1_ESM.docx]

**Supplementary materials**


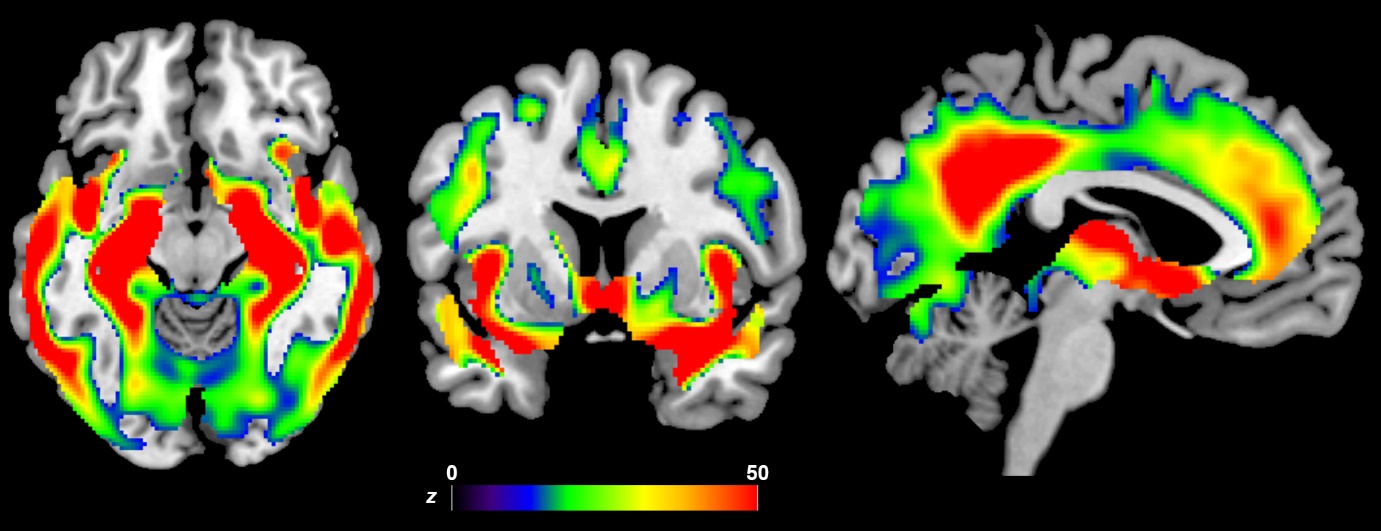


**Supplementary Figure 1.** Results of the VBM ANOVA model comparing GM volume across CU, MCI and AD groups (*n* = 800; *p* < 0.05 FWE-corrected for multiple comparisons).


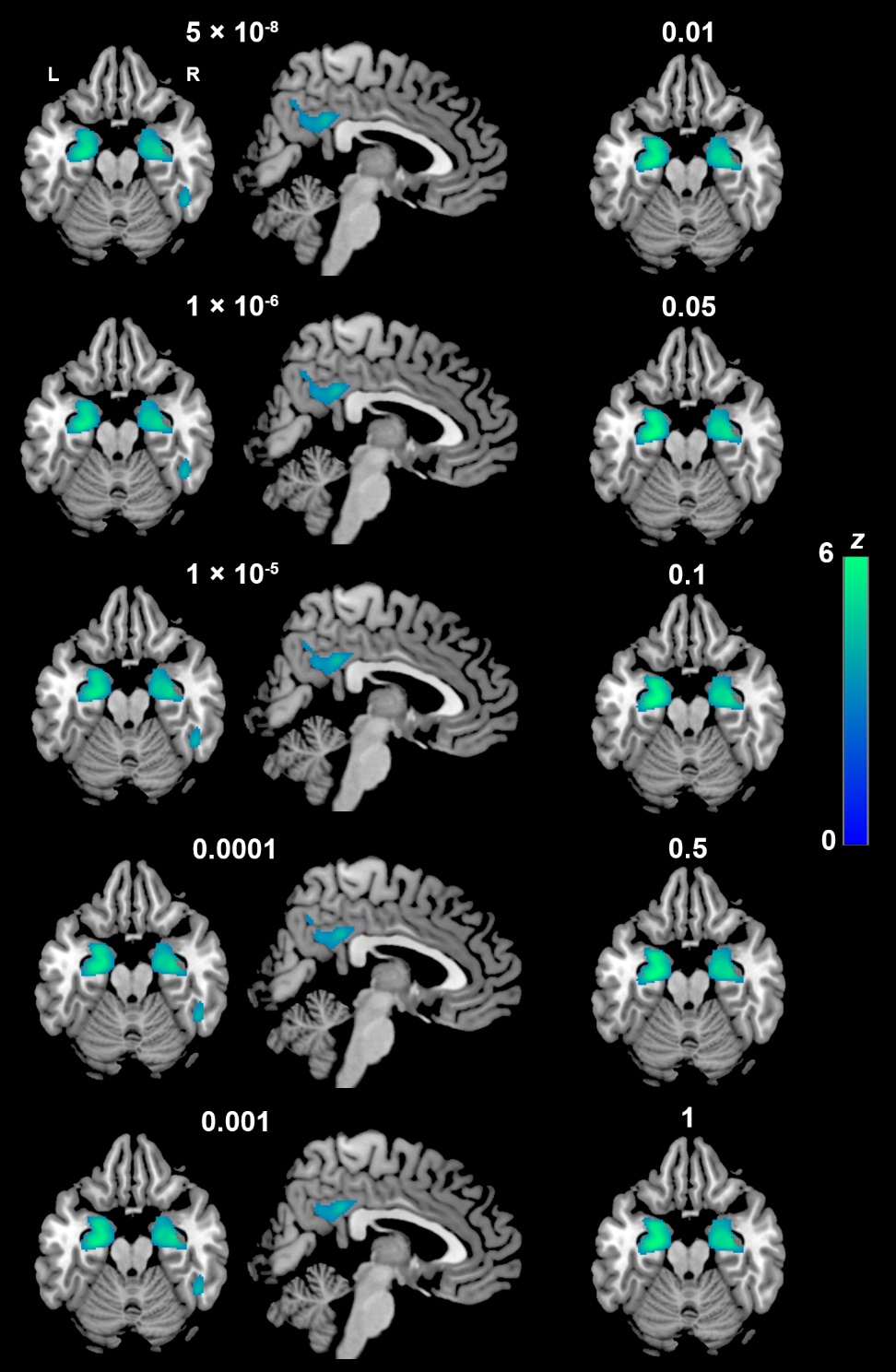


**Supplementary Figure 2.** Voxel-based negative associations between AD-PRS (calculated using 10 thresholds) and GM volume in the whole sample (*n* = 800; *p* < 0.05 FWE-corrected for multiple comparisons).


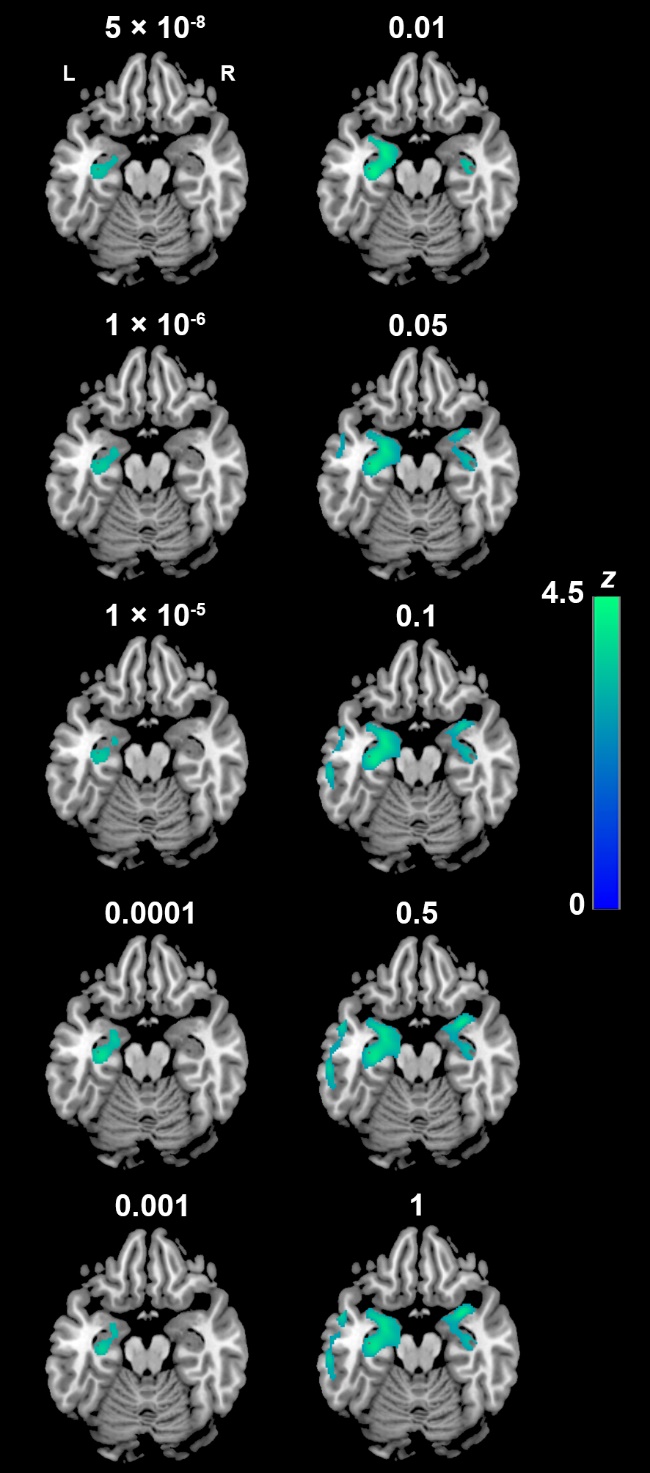


**Supplementary Figure 3.** Voxel-based negative associations between AD-PRS_noAPOE_ (calculated using 10 thresholds) and GM volume in the whole sample (*n* = 800; *p* < 0.05 FWE-corrected for multiple comparisons).


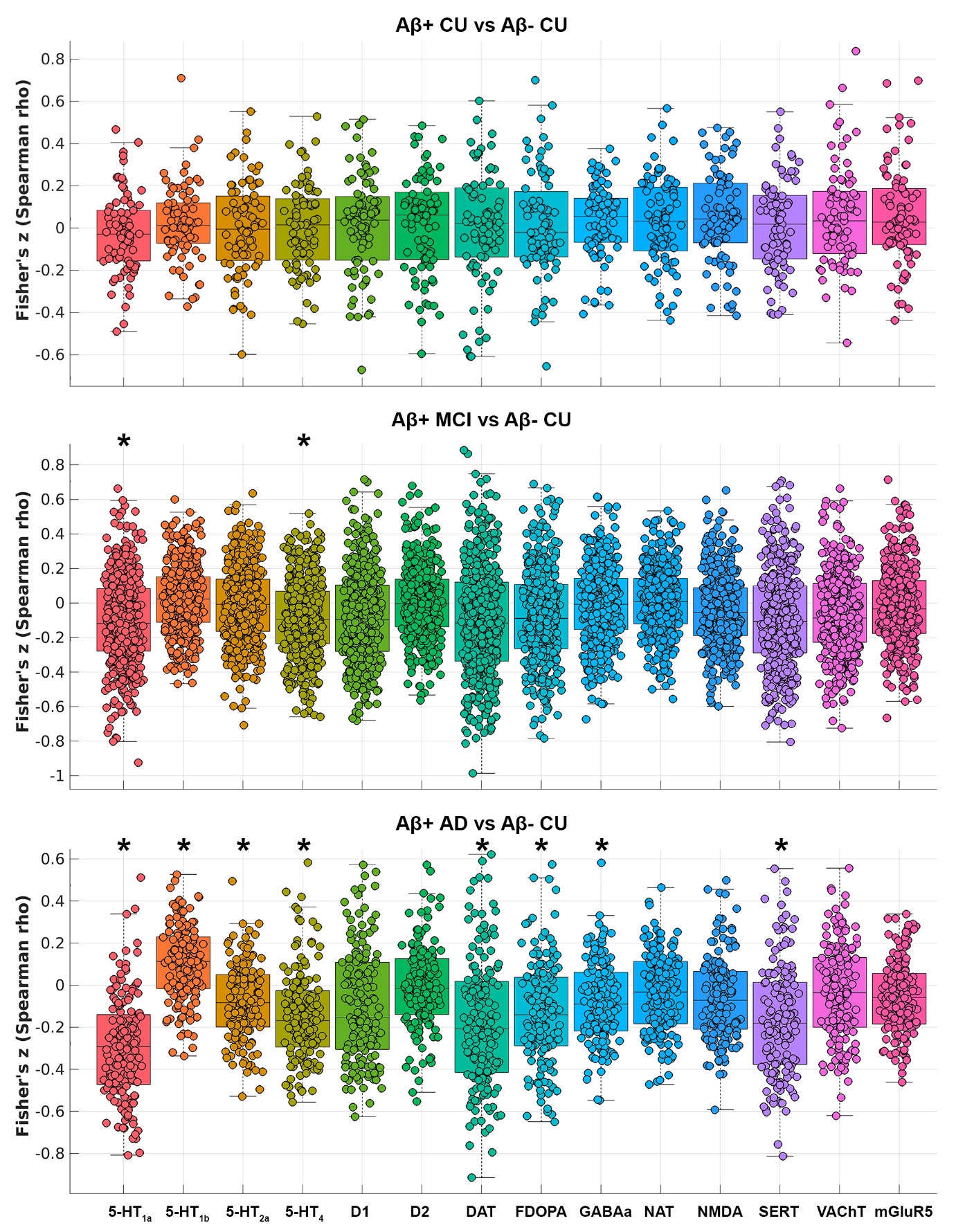


**Supplementary Figure 4.** Associations between grey matter atrophy in Aβ+ CU, Aβ+ MCI and Aβ+ AD groups and *a priori* density values of neurotransmitters compared with Aβ- CU participants (significant results are indicated by an asterisk).


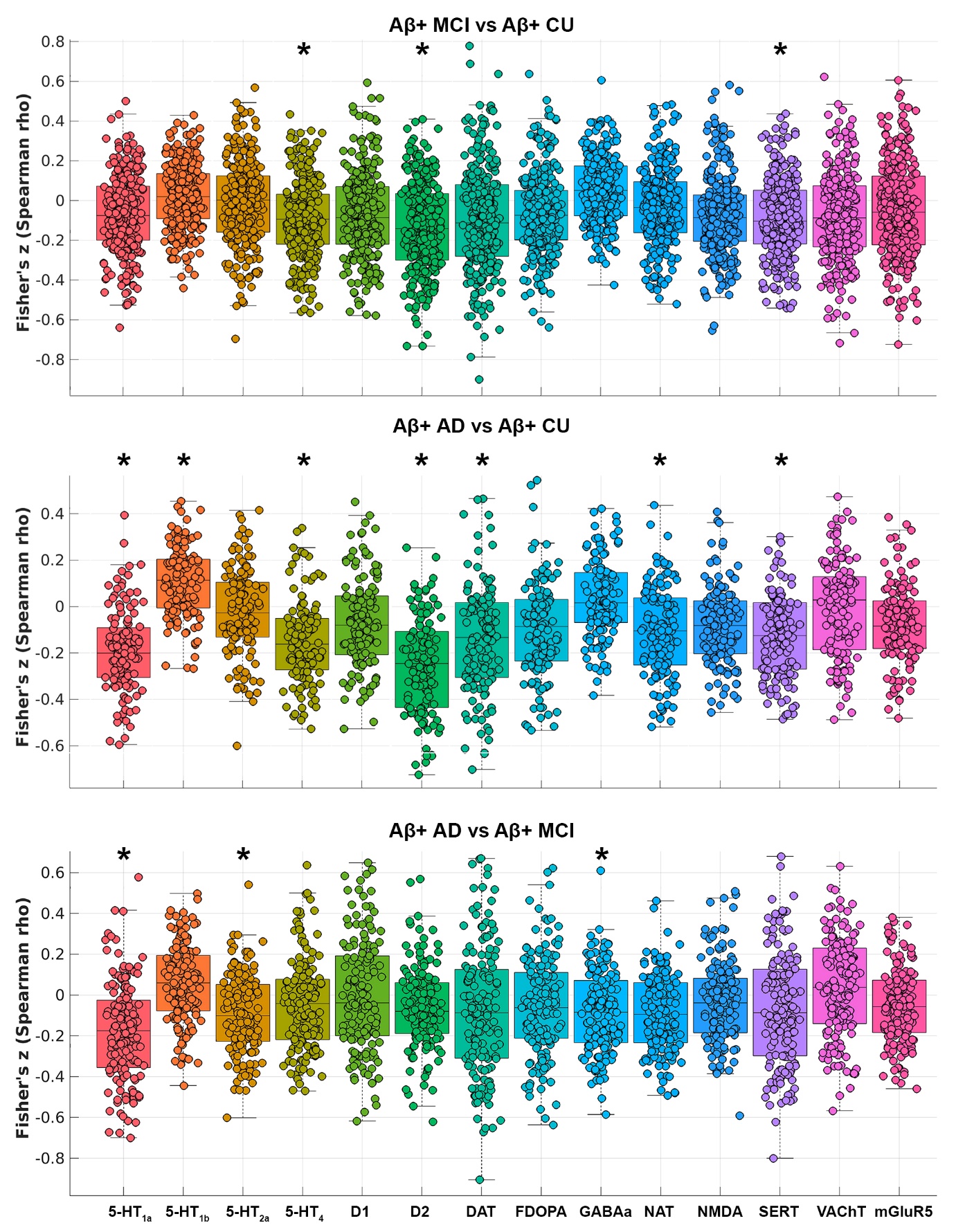


**Supplementary Figure 5.** Associations between grey matter atrophy in Aβ+ groups and *a priori* density values of neurotransmitters (significant results are indicated by an asterisk).


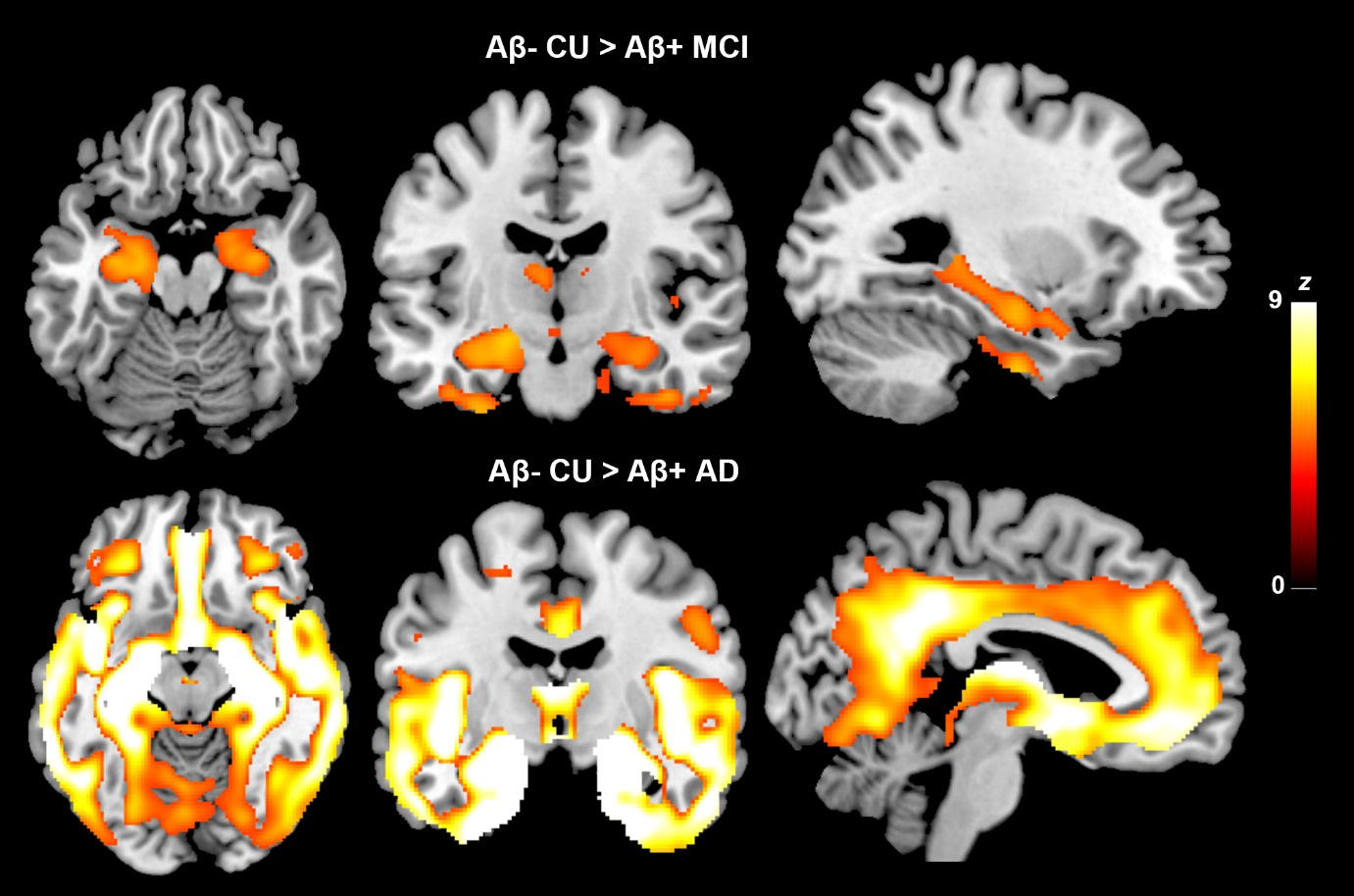


**Supplementary Figure 6.** Results of the VBM analyses comparing Aβ+ patients with Aβ- CU participants (*p* < 0.05 FWE-corrected for multiple comparisons).


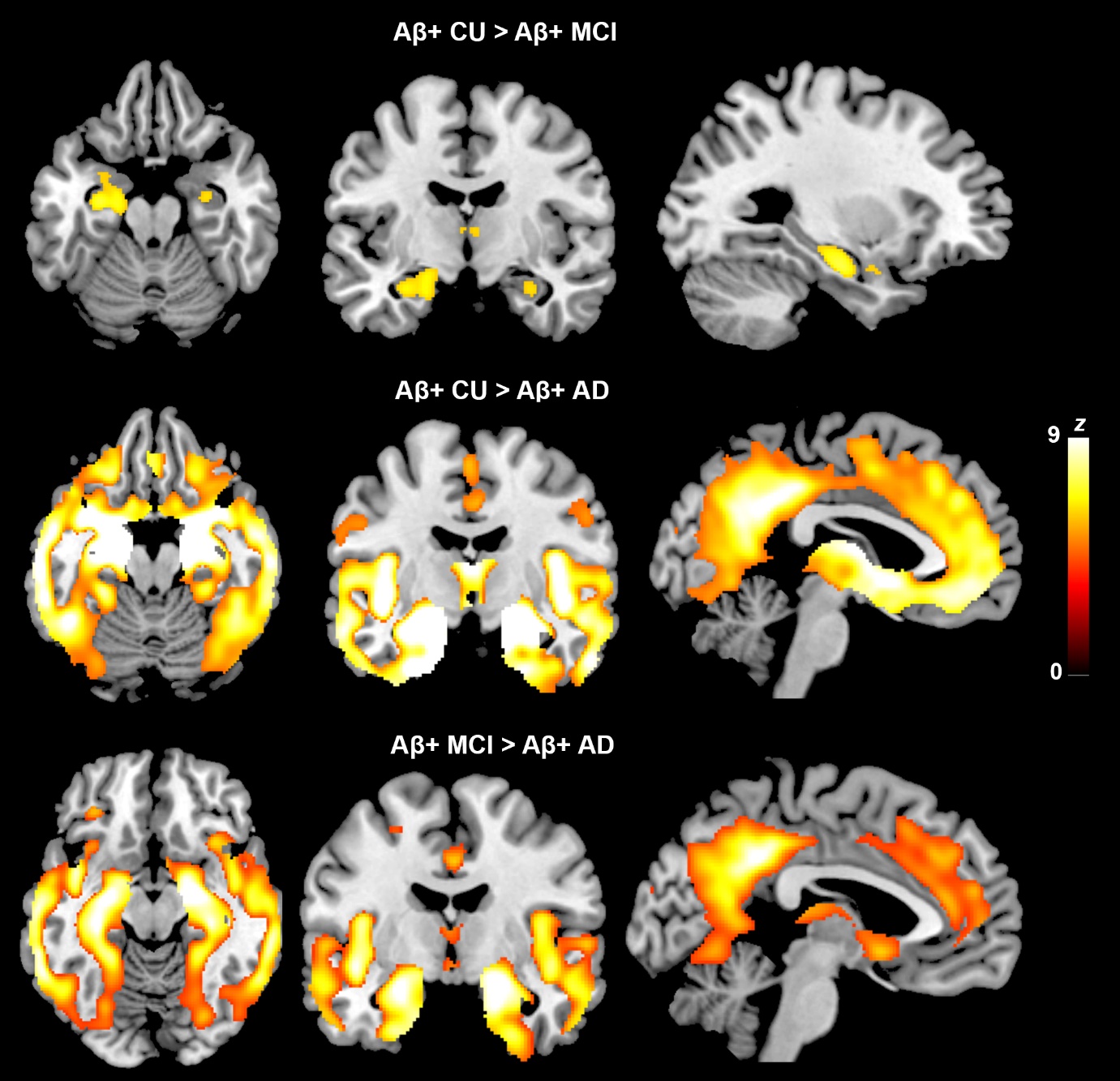


**Supplementary Figure 7.** Results of the VBM analyses comparing all Aβ+ groups (*p* < 0.05 FWE-corrected for multiple comparisons).


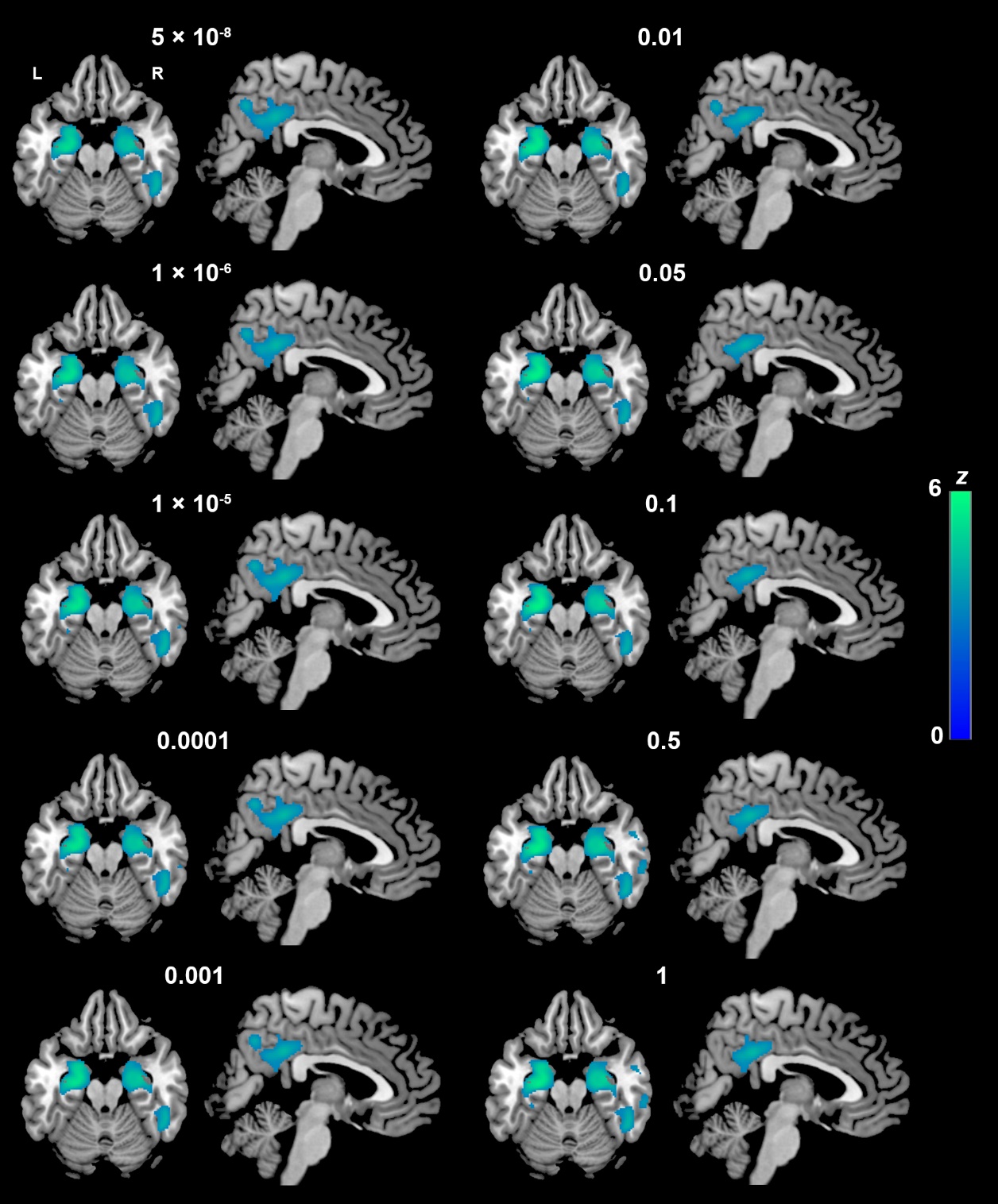


**Supplementary Figure 8.** Voxel-based negative associations between AD-PRS (calculated using 10 thresholds) and GM volume in the Aβ+ sample (*n* = 481; *p* < 0.05 FWE-corrected for multiple comparisons).

**Supplementary Table 1.** Demographic and clinical profile of Aβ+ and Aβ- CU participants. All summary statistics are mean (standard deviation) unless otherwise specified (Bonferroni-corrected *p* = 0.0042).

| **Variables** | **Aβ- CU (*n* = 105)** | **Aβ+ CU (*n* = 79)** | **Aβ+ MCI (*n* = 274)** | **Aβ+ AD (*n* = 128)** | ***F*** | ***p*** |
| --- | --- | --- | --- | --- | --- | --- |
| *Demographic* |  |  |  |  |  |  |
| Age | 73.8 (5.9) | 73.6 (5.8) | 74.4 (7.9) | 76.1 (7.9) | 10.8^a^ | 0.012 |
| Education | 16.6 (2.8) | 16.3 (2.4) | 16.1 (2.8) | 15.9 (2.9) | 4.4^a^ | 0.220 |
| Sex (%, F/M) | 51.4/48.6 | 53.2/46.8 | 39.8/60.2 | 45.3/54.7 | 6.9^b^ | 0.075 |
| *Cognitive* |  |  |  |  |  |  |
| MMSE (total score) | 29.1 (1.1)^c,d^ | 29.1 (1.3)^c,d^ | 27.7 (2.0)^d^ | 21.2 (5.1) | 280.4^a^ | <0.001 |
| CDT (Drawing) | 4.7 (0.5)^c,d^ | 4.7 (0.5)^d^ | 4.6 (0.8)^d^ | 3.2 (1.5) | 111.8^a^ | <0.001 |
| CDT (Copy) | 4.9 (0.4)^d^ | 4.9 (0.3)^d^ | 4.7 (0.5)^d^ | 4.0 (1.3) | 70.8^a^ | <0.001 |
| LMT (Immediate recall) | 14.7 (3.0)^c,d^ | 14.8 (2.9)^c,d^ | 9.6 (3.9)^d^ | 4.0 (3.1) | 331.2^a^ | <0.001 |
| LMT (Delayed recall) | 13.8 (3.3)^c,d^ | 13.6 (3.1)^c,d^ | 7.3 (4.0)^d^ | 1.6 (2.8) | 374.5^a^ | <0.001 |
| CFT (Animals) | 20.7 (5.5)^c,d^ | 21.9 (5.6)^c,d^ | 17.8 (5.0)^d^ | 10.7 (5.2) | 198.4^a^ | <0.001 |
| TMT-A (seconds) | 32.6 (9.8)^c,d^ | 34.0 (10.5)^c,d^ | 40.1 (16.9)^d^ | 69.6 (45.6) | 97.7^a^ | <0.001 |
| *Imaging and biomarkers* |  |  |  |  |  |  |
| TIV | 1444.6 (140.1) | 1417.4 (129.3) | 1469.0 (143.1) | 1449.5 (162.0) | 7.3 | 0.063 |
| APOE (%) |  |  |  |  | 108.9^b^ | <0.001 |
| *ε4ε4* | 0.0^c,d,e^ | 3.8^d^ | 9.1^d^ | 20.3 |  |  |
| *ε3ε4* | 15.2^c,d,e^ | 30.4^d^ | 39.4^d^ | 51.5 |  |  |
| *ε3ε3* | 65.7^c,d^ | 57.0^c,d^ | 42.3^d^ | 23.4 |  |  |
| *ε2ε4* | 1.0 | 0.0 | 3.6 | 3.1 |  |  |
| *ε2ε3* | 18.1^c,d^ | 8.9^d^ | 5.1 | 1.6 |  |  |
| *ε2ε2* | 0.0 | 0.0 | 0.4 | 0.0 |  |  |

Aβ: Amyloid beta, AD: Alzheimer’s disease, APOE: Apolipoprotein E, CDT: Clock Drawing test, CFT: Category Fluency test, CU: Cognitively unimpaired, F: Female, LMT: Logical Memory test, M: Males, MCI: Mild cognitive impairment, MMSE: Mini Mental State Examination, TMT-A: Trail Making Test – part A

^a^ Kruskal-Wallis test

^b^ χ^2^ test

^c^ Significant difference compared with Aβ+ MCI group

^d^ Significant difference compared with Aβ+ AD group

^e^ Significant difference compared with Aβ+ CU group

**Supplementary Table 2.** Comparisons (Kruskall-Wallis test) of demographic and clinical profiles across MCI groups stratified by amyloid status. All summary statistics are mean (standard deviation) unless otherwise specified (Bonferroni-corrected p = 0.05/12 = 0.0042).

| **Variables** | **Aβ− MCI (*n* = 148)** | **Aβ+ MCI (*n* = 274)** | **Aβ Unknown MCI (*n* = 20)** | **χ^2^** | ***p*** |
| --- | --- | --- | --- | --- | --- |
| *Demographic* |  |  |  |  |  |
| Age | **71.3 (8.3)** | **74.4 (7.6)^a^** | **73.6 (7.5)** | **14.86** | **<0.001** |
| Education | 15.8 (2.6) | 16.1 (2.8) | 15.6 (3.6) | 1.15 | 0.563 |
| Sex (%, F/M) | 45.9/54.1 | 39.8/60.2 | 30.0/70.0 | 2.62^b^ | 0.269 |
| *Cognitive* |  |  |  |  |  |
| MMSE (total score) | 28.4 (1.5) | 27.7 (2.0) | 27.9 (1.6) | 9.77 | 0.008 |
| CDT (Drawing) | 4.6 (0.6) | 4.5 (0.8) | 4.6 (0.6) | 3.16 | 0.206 |
| CDT (Copy) | 4.8 (0.4) | 4.7 (0.5) | 4.9 (0.3) | 4.07 | 0.131 |
| LMT (Immediate recall) | 10.6 (3.2) | 9.6 (3.9) | 10.2 (4.3) | 7.98 | 0.018 |
| LMT (Delayed recall) | **8.4 (2.9)** | **7.3 (4.0)^a^** | **6.9 (4.8)** | **12.51** | **0.002** |
| CFT (Animals) | 18.8 (4.9) | 17.8 (5.0) | 18.9 (5.7) | 4.46 | 0.108 |
| TMT-A (seconds) | 32.3 (11.9) | 40.1 (16.9) | 36.8 (12.7) | 8.24 | 0.016 |
| *Biomarkers and imaging* |  |  |  |  |  |
| TIV | 1440.4 (129.0) | 1469.0 (143.1) | 1468.0 (124.6) | 3.34 | 0.188 |
| APOE ε4 (%, C/NC) | **17.6/82.4** | **52.2/47.8^a^** | **30.0/70.0** | **49.00** | **<0.001** |

Aβ: Amyloid beta, AD: Alzheimer’s disease, APOE: Apolipoprotein E, C: Carriers, CDT: Clock Drawing test, CFT: Category Fluency test, CU: Cognitively unimpaired, F: Female, LMT: Logical Memory test, M: Males, MCI: Mild cognitive impairment, MMSE: Mini Mental State Examination, NC: Non-carriers, TMT-A: Trail Making Test – part A

^a^ Significantly different from the Aβ− MCI group

^b^ Chi-square test

**Supplementary Table 3.** Comparisons (Kruskall-Wallis test) of demographic and clinical profiles across AD groups stratified by amyloid status. All summary statistics are mean (standard deviation) unless otherwise specified (Bonferroni-corrected p = 0.05/12 = 0.0042).

| **Variables** | **Aβ− AD (*n* = 13)** | **Aβ+ AD (*n* = 128)** | **Aβ Unknown AD (*n* = 14)** | **χ^2^** | ***p*** |
| --- | --- | --- | --- | --- | --- |
| *Demographic* |  |  |  |  |  |
| Age | 80.8 (8.3) | 76.1 (7.9) | 80.5 (6.4) | 7.65 | 0.022 |
| Education | 16.5 (2.5) | 15.9 (2.9) | 16.3 (3.0) | 0.67 | 0.714 |
| Sex (%, F/M) | 69.2/30.8 | 54.7/45.3 | 50.0/50.0 | 1.20 | 0.549 |
| *Cognitive* |  |  |  |  |  |
| MMSE (total score) | 24.9 (2.5) | 21.2 (5.1) | 19.9 (5.0) | 10.24 | 0.006 |
| CDT (Drawing) | 4.0 (1.2) | 3.2 (1.5) | 2.9 (1.6) | 4.06 | 0.131 |
| CDT (Copy) | 4.5 (0.9) | 4.0 (1.3) | 3.9 (1.2) | 2.76 | 0.252 |
| LMT (Immediate recall) | 7.1 (5.1) | 4.0 (3.1) | 3.7 (2.8) | 4.92 | 0.086 |
| LMT (Delayed recall) | 4.9 (5.3) | 1.6 (2.8) | 1.5 (2.0) | 5.60 | 0.061 |
| CFT (Animals) | 13.5 (4.4) | 10.7 (5.2) | 11.2 (5.2) | 3.59 | 0.166 |
| TMT-A (seconds) | 45.9 (18.5) | 69.9 (45.6) | 73.6 (40.0) | 3.74 | 0.154 |
| *Biomarkers and imaging* |  |  |  |  |  |
| TIV | 1545.2 (214.6) | 1449.5 (162.0) | 1412.7 (126.0) | 3.02 | 0.221 |
| APOE ε4 (%, C/NC) | **7.7/92.3** | **75.0/25.0^a^** | **78.6/21.4^a^** | **25.88^b^** | **<0.001** |

Aβ: Amyloid beta, AD: Alzheimer’s disease, APOE: Apolipoprotein E, C: Carriers, CDT: Clock Drawing test, CFT: Category Fluency test, CU: Cognitively unimpaired, F: Female, LMT: Logical Memory test, M: Males, MCI: Mild cognitive impairment, MMSE: Mini Mental State Examination, NC: Non-carriers, TMT-A: Trail Making Test – part A

^a^ Significantly different from the Aβ− AD group

^b^ Chi-square test
